# Progress in Fevers

**Published:** 1902-02-22

**Authors:** 


### Progress in Fevers.

(*Continued from page 343.*)

**Tetanus.**—Josias has recently performed some experimental work on goats,[13] with a view to testing the efficacy of the treatment described by Bacelli at the Italian Congress of Medicine in 1888. Four goats were injected subcutaneously with lethal doses of tetanus toxin. As soon as symptoms appeared, three of the goats received one or more subcutaneous injections of a 2 per cent. solution of carbolic acid. All four animals died after four or five days, having shown the usual phenomena of tetanus. The control goat, the one not treated with carbolic acid, although more severely ill at first, lived the longest. A repetition of the experiment, using weaker carbolic acid, gave a similar result. Josias therefore concludes that Bacelli's treatment in tetanus is useless. E. M. Sympson[14] records a case of recovery from tetanus in which 50 c.c. of antitetanic serum were injected. Potassium bromide and chloral were also given. The patient, a man aged 45, showed symptoms on the eleventh day; treatment was begun on the fourteenth day. Several days after the spasms had ceased, fever with pain and swelling of the legs occurred, but yielded ultimately to salicylate of quinine. The complication was regarded as rheumatic. T. G. Scott[15] reports a fatal case, in which 30 c.c. of antitoxin were employed in conjunction with morphia and chloroform inhalations. The patient, a woman aged 21, showed symptoms on the fifth day. The wound on the foot inflamed and was freely excised. The patient sank exhausted and cyanosed on the seventh day. E. Mackey[16] reports three cases, all males, successfully treated by him with serum. The incubation periods were about 14 days in two cases, 10 days in the third. Of serum, 116, 131, and 105 c.c. were injected respectively. Sedatives—chloral, bromide, and morphia—were used in moderation. The symptoms showed an intermittent and recrudescent character, and sometimes were temporarily increased by the injections, although relieved later, and tending to recur on suspension of the treatment. Dangerous collapse appeared several times, especially in one case. One case showed an urticarial rash, and another developed a red rash over joints, body and face, with pain in one leg. For determining the value of antitetanic serum certain facts are of importance in recording cases,[17] *e.g.* accurate and, if possible, bacteriological diagnosis; time of incubation; source, dose, and time of administration of the serum. The concurrent use of sedatives is important, and the proper cleansing, or, if desirable, amputation of the original wound area imperative, since the bacillus of tetanus lives only in the wound, not in the blood, and only the toxins are absorbed into the circulation.

**Yellow Fever.**—The researches of W. Reed, of the U.S.A. Army, and his colleagues in Cuba,[18] into the nature and cause of yellow fever, were begun for the purpose of verifying or disputing the claims of Sanarelli's *bacillus icteroides* to be the causative agent, and ended in proving the mosquito to be the carrier of infection. Out of 32 cases in all examined, in none was the bacillus found, nor could any bacterium be cultivated from the blood by aërobic methods before or after death. Finlay had previously suggested, in 1881, that the mosquito propagated yellow fever. Most of Reed's researches were carried out in a specially constructed camp—Camp Lazear, in memory of his co-worker Lazear—one mile from Quemados, Cuba, with strict scientific precautions. The subjects of experiment were men, Americans or Spaniards, who for love of glory or of gain volunteered for, or consented to, the experiment. No fatal case occurred. The experiments proved three main facts:—(*a*) Mosquitoes, previously fed on the blood of yellow-fever patients, were allowed to bite non-immune individuals who had been rigidly isolated for many days. Infection followed in 10 cases out of 13. (*b*) No infection seemed to arise from fomites. For an average of 21 nights each 7 men slept in a purposely ill-ventilated and sunless room, using bedding and clothes soiled by the discharges of yellow-fever patients. Not one developed the disease. One of these men subsequently allowed himself to be bitten by three mosquitoes fed 39 days previously on a yellow-fever patient. In 3 to 4 days he developed the disease. (*c*) Yellow fever followed the subcutaneous injection of blood from a yellow-fever patient into non-immune isolated individuals. The incubation period was generally 3½ to 4 days. It appears that a certain time must elapse—apparently about 12 days in summer and 18 or more in winter—before the bite of the infected mosquito can produce the disease. One man bitten by "4th-day" and again by "11th-day" insects remained free, but developed an attack after being stung by "17th-day" insects. One man also was infected by a "57th-day" insect. The former of these cases disproves Finlay's theory that the bite of a contaminated insect secures protection, even though no attacks follows. W. Reed and J. Carroll[19] describe and figure the mosquito *Stegomyia fasciata*, which is believed to be concerned in the propagation of the disease. This insect was originally known as *Culex fasciatus*, but quite recently it has been transferred by Theobald from the genus *Culex* to a new genus, *Stegomyia*.[20] It is the only species known at present as able to act intermediate host to the specific organism of yellow fever. The mosquito is a handsome insect, with a broad semi-lunar stripe on the lateral surface of the thorax, and white stripes at the bases of the tarsal joints. As in *Culex*, the palpi are short in the female. It is widely distributed over hot countries at low altitudes, and breeds in any collection of still water, not being deterred by the presence of some fæcal matter. The eggs, 20 to 75 in number, jet-black and somewhat cylindrical, are deposited on water at night singly or in small groups. If submerged they do not rise again, but hatch below the surface. They are very resistent to drying and to freezing, and have hatched after being kept dried for 3 months. The incubation period generally takes about 3 days, the larval stage 7 days, and the pupal stage 2 days. The larva and pupa resemble those of *culex*. The eggs do not hatch below a continuous temperature of 68° F. (20° C.). The female, if impregnated, will suck blood on the 2nd or 3rd day of existence by day as well as by night, and at a temperature as low as 62° F., but probably not lower. This fact will explain the seasonal prevalence of yellow fever in different places. It probably takes 12 days or more for the infective organism to pass through the body of the mosquito and reach the salivary glands. This interval, plus the incubation period of the disease, 3 to 4 days, corresponds to the time, 2 to 3 weeks, generally observed to elapse between one case of yellow fever and the first secondary case. Infectivity probably coincides with the life of the infected insect. Major R. Ross[21] considers that the mosquito-borne theory of yellow fever, enunciated by Reed, Carroll and Agramonte, is beyond dispute. It is possible that the as yet unknown pathogenic organism may prove to be of vegetable origin, and there is nothing essentially improbable in the idea of gnats becoming infected with schizomycetes, and through their salivary glands, or otherwise, passing on the organism to other hosts. Since the publication of the results obtained by Reed and his colleagues evidence is accumulating to show that the propagation of yellow fever by personal contact and by fomites has long been doubted by many physicians. A. H. Doty, Health Officer for the Port of New York,[22] quotes views to this end expressed by several authorities about 1798 and 1806. In 1859 in New York City a resolution was passed by a convention of sanitarians declaring that personal quarantine might safely be abolished, provided fomites were rigidly disinfected. Dr. Doty, five years ago, put in force at New York Port regulations in accordance with his own disbelief in infection by personal contact or by fomites. If vessels five days or more out from an infected port arrive with "all well," no one is detained, although disinfecting is performed in accordance with existing quarantine regulations. Five days is believed to be the maximum incubation period. Although it seems highly probable that vessels lying off infected ports should become infested with infected mosquitoes, actually this seems extremely rare. The insects appear to keep near to their breeding-places. Doty advises that health officers should study mosquitoes, and devise means for protecting yellow-fever patients from mosquito bites; that vessels five days out from an infected port should be accounted free; and that vessels from nearer ports should be accounted free after the five days are completed, provided in each case that a very careful search leads to the detection of no case of illness on board. W. C. Gorgas,[23] Medical Officer of Havana, describes the precautions adopted for checking the disease in that city since February last, when the researches of the Army Medical Board became known. Previously the patient was strictly quarantined, and the room subsequently disinfected with formalin. Now the patient selects his rooms, which are, generally within two hours of notification, fitted with wire-gauze windows and doors. Admittance with strict precautions is granted only to a few immune persons. At the same time mosquitoes in the same and surrounding houses are destroyed by burning pyrethrum powder, 1 lb. to 1,000 cubic feet of air space. Attention is also given to the general destruction of mosquitoes. A numerous staff of inspectors visits the whole town once a month; cesspools, water-tanks, and other receptacles are rendered mosquito-proof, or treated with crude coal oil every two weeks. So far the diminution in the disease is very satisfactory. Surgeon-Major Havard, stationed at Havana,[24] explains how Caldas and Bellinzaghi were given an opportunity, in that city, of demonstrating the value of their so-called prophylactic serum and vaccine. The demonstration entirely failed, and the Commission appointed to examine into it decided that Caldas's vaccine, prepared from an intestinal microbe, was utterly useless as a prophylactic against yellow fever, and they discouraged further experiment.

[13] Lancet, Nov. 2, p. 1234. [14] Lancet, Sept. 14, p. 729. [15] Lancet, Oct. 19, p. 1040. [16] Lancet, Nov. 9. [17] Ibid. [18] Med. Rec., Aug. 10. [19] Med. Rec., Oct. 26. [20] Ibid., Aug. 10. [21] Brit. Med. Jour., Sept. 7. [22] Med. Rec., Oct. 26. [25] Med. Rec., Sept. 7. [24] Med. Rec., Sept. 14.

